# Bondonic Effects in Group-IV Honeycomb Nanoribbons with Stone-Wales Topological Defects

**DOI:** 10.3390/molecules19044157

**Published:** 2014-04-03

**Authors:** Mihai V. Putz, Ottorino Ori

**Affiliations:** 1 Laboratory of Computational and Structural Physical-Chemistry for Nanosciences and QSAR, Biology-Chemistry Department, Faculty of Chemistry, Biology, Geography, West University of Timişoara, Pestalozzi Street No.16, Timişoara, RO-300115, Romania; 2 Actinium Chemical Research, Via Casilina 1626/A, Rome 00133, Italy; E-Mail: ottorino.ori@alice.it

**Keywords:** bondons, electronegativity, graphene, silicene, germanene, phase transition, 4th order quantum propagator

## Abstract

This work advances the modeling of bondonic effects on graphenic and honeycomb structures, with an original two-fold generalization: (i) by employing the fourth order path integral bondonic formalism in considering the high order derivatives of the Wiener topological potential of those 1D systems; and (ii) by modeling a class of honeycomb defective structures starting from graphene, the carbon-based reference case, and then generalizing the treatment to Si (silicene), Ge (germanene), Sn (stannene) by using the fermionic two-degenerate statistical states function in terms of electronegativity. The honeycomb nanostructures present *η*-sized Stone-Wales topological defects, the isomeric dislocation dipoles originally called by authors Stone-Wales wave or SWw. For these defective nanoribbons the bondonic formalism foresees a specific phase-transition whose critical behavior shows typical bondonic fast critical time and bonding energies. The quantum transition of the ideal-to-defect structural transformations is fully described by computing the caloric capacities for nanostructures triggered by *η*-sized topological isomerisations. Present model may be easily applied to hetero-combinations of Group-IV elements like C-Si, C-Ge, C-Sn, Si-Ge, Si-Sn, Ge-Sn.

## 1. Introduction

With the irresistible rise of graphene, great attention has been paid by the scientific community to the spectacular properties of this carbon monolayer, the “Nobel prized” new carbon allotrope which ‒ a decade after its discovery in 2004 [1]—still promises innovative *technological* solutions for many issues in physics and nanotechnology, but clearly, the real *breakthrough discovery* initiating the *golden-age* of graphene is still missing [2]. This remains an unachieved goal, a severe scientific challenge that pushes experimentalists worldwide to solve the barriers, both technological and cost-wise, which hinder mass-applications of this *one-atom-thick fabric of carbon* with its “extreme” mechanical and electronic features. The risk of a frustrating record with no application results for grapheme is the same fate which (somehow unexpectedly) prevented so far any practical C*_n_* fullerene applications, has been recently denied by the most authoritative review on the subject [3], waiting for a “manufacturing” turning-point; these authors in fact repute the fact that graphene will eventually become attractive for industrial applications providing that “mass-produced graphene will guarantee the same performances as the best samples obtained in research laboratories”.

From a general perspective, ten years of investigations on graphenic honeycomb lattices point out the scientific relevance of *monolayer materials* like hexagonal BN, MoS_2_ and others, whose 2D crystals present a rich diversity of physico-chemical properties that can be further specialized by combining variable stacks of heterostructures (often called *van der Waals heterostructures* due to the presence of van der Waals-like forces gluing the layers together [4] as in normal graphite crystals) with applications, for example, in vertical tunneling transistors [5]. It is however commonly accepted [3] that at least for microprocessors, graphene-based logic elements will replace the silicon technology only after 2025, the main physical limit being so far represented by the reduced value of the induced bandgap in graphene still limited to 360 meV with a reduction of performances of a factor 10^3^ if compared to current silicon devices. This impasse is one of the main reasons focusing research today on a movement from *carbon-based* toward *silicon-based hexagonal systems* in microelectronics.

The natural candidate for such a class of material is *silicene*, the honeycomb monolayer theoretically introduced as the all-silicon version of graphene [6,7], which has been synthesized by chemical exfoliation of calcium disilicide resulting in silicon 2D nanosheets of 0.37 nm thickness [8] or, more recently, by epitaxial growth on metallic surfaces. Topologically, silicene, Si-NR and graphene share the same kind of hexagonal mesh made of 3-connected atoms, the main distinguishing character being for silicene the structural distortion (see the next section). The topology of the honeycomb lattices allows the creation of *isomeric* defects consisting in a double pair of 5|7 rings, the so-called Stone-Wales rotation or SW topological defect, an important structural change which modifies the band configurations for such low-dimensional systems. In fact, sparse SW rotations immediately open a band gap of 0.1 eV in silicene fragments.

In this context therefore a comprehensive treatment of the electron behavior in nanostructures made of Group-IV elements is highly necessary and represents in fact the main scope of this work. Our original approach is based on the properties of the *bondon*, the recently introduced *quasi-particle* arising from the Bohmian quantum description of the matter [9,10,11,12,13,14,15,16] which is the expression of the *quantization of the chemical bond* by *bosonation* of the electronic-pairs [11,15]. High order path integral formalism and *topological potentials* are also used here as basic theoretical tools (see Section 3). The main outcome of the current study consists in the original prediction of a specific *phase* *transition* induced by the bondonic movements in a 1D honeycomb system rearranged by SW topological defects; the method clearly discriminates various chemical nanostructures, e.g., graphenic (C-based), silicenic (Si-based), germanenic (Ge-based) and stannenic (Sn-based) nanoribbons, by using the appropriate electronegativity functions. Future experimental and theoretical works will assess the general validity of the reported theoretical conclusions, allowing a deeper description of the *bondonic chemistry* of Group-IV elements at the nanoscale.

## 2. Structure and Topology of Honeycomb Nanoribbons

**Figure 1:**
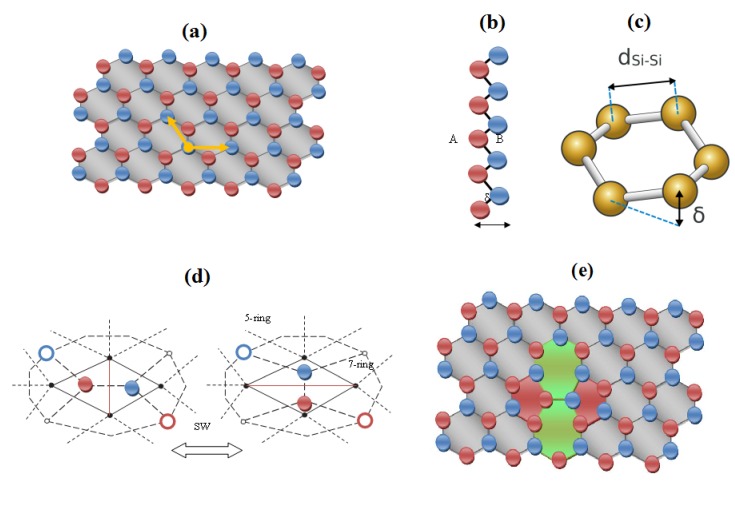
(**a**) The honeycomb mesh characterizing the 1D nanoribbons with the two independent atoms and the two unit cell vectors which, by translation, cover the entire structure; (**b**) side view of the lattice and (**c**) of the buckling structural parameter δ spacing silicene hexagonal sublattices A and B; the Si-Si bond distance is also depicted; in silicene typical distortion parameter is δ=0.44 Ȧ with *d*(Si-Si)=2.25 Ȧ; (**d**) The 5|7|7|5 Stone-Wales rotation seen in the direct and dual representation in the nanoribbon honeycomb mesh; (**e**) view of the SW defect in the mesh (**a**), pentagonal (heptagonal) rings are in red (green).

In this section the main characteristics of 1D nanoribbons made of Group-IV elements are briefly presented. Like graphene, these systems exhibit the genuine honeycomb structure given in Figure 1a. For silicene in particular, deposition techniques under ultra-high vacuum conditions on silver (110) plane guide the production [17] of one-atom-thick metallic Si nanowires (or silicon nanoribbons Si-NR). Metallic Si-NR are suitable for being promoted to *n* or *p*-type semiconductors by chemical doping.

Such extended Si nanoribbons are built by the action of the two-components *2p* quantum levels, corresponding to the electronic contributions coming from the two distinct silicon atomic sites. Experimental and theoretical investigations by scanning tunneling microscopy (STM) and *ab initio* calculations based on density functional theory (DFT) [18,19] state that silicene 1D stripes grow on the silver substrate with a length exceeding 100 nm and with 1.6 nm “magic” width. Si nanowires are moreover able to reach a “self-organized”, regular coverage on the substrate surface with a spacing of about 2 nm [19]. These important structural results have been consecrated [20] by the same team of researchers who succeeded in the epitaxial formation of silicene 2D sheets on a silver (111) substrate. By mean of scanning tunneling microscopy and angular-resolved photoemission spectroscopy measures, in conjunction with DFT simulations, the study ultimately confirms the silicene *buckled* honeycomb arrangement (Figure 1b). The *buckling* distortion δ (Figure 1c) moves the system out from perfect (graphene) planarity. Such a chair-like puckering of the Si 6-rings corresponds to a *buckling* parameter of δ = 0.44 Ȧ with a bond length of *d*(Si-Si) = 2.25 Ȧ [21] whereas in graphene the inter-atomic distance is *d*(C-C) = 1.42 Ȧ.

The chemical stability of buckled honeycomb structures is substantially granted by the “puckering induced” dehybridization-effect which allows *p_z_* orbitals, oriented normally to the layer, making linear combinations with the *s* orbitals, forming π bonding and hence π and π* bands, similarly to graphene case. Comparative values for the based on the modified Harrison bond orbital method are reported in [22] resulting in an atomic binding energy of *E_atom_* = 13.5 eV and *E_atom_* = 7.1 eV for graphene and silicene respectively. It is worth noticing that, from the structural point of view, the two independent atoms that constitute the graphene unit cell generate in silicene the two distinct sublattices A and B, laying in two δ-spaced parallel planes (Figure 1b) [23].

*Topological mechanisms* enrich the physico-chemical features of the honeycomb lattices by creating isomeric transformations, respecting both the number of atoms and the number of bonds. Such a particular one-bond rotation, the so-called Stone-Wales (SW) rotation or SW topological defect, transforms four hexagons in a double pair of 5|7 rings (Figure 1d), changing the band configurations of the honeycomb mesh. According to recent Monte-Carlo simulations on the subject [24], this mechanism alters the long-range planarity of the graphenic layer over regions with the size of many nanometers, reaching large out-of-plane deformations δ ≈ 1.7 Ȧ. These *buckled SW transformations* may have therefore a possible role during fullerene and nanotube formation. Figure 1e shows the characteristic heptagon-pentagon double pair appearing in the hexagonal network after a SW rotation. The generation of SW defects *solely* depends from the 3-connectivity of the lattice atoms. SW-compatible patterns reflect the properties of the topological adjacency matrix of the system. Typical values for the energy barrier *E_b_* opposing such a SW rotation in graphene [24] and Ag(111)-grown silicene [25] are *E_b_* ≈ 5 eV and *E_b_* ≈ 2.8 eV, respectively. This large difference reflects the basic structural fact that silicene has a larger inter-atomic distance compared to graphitic layers, so an easier formation of topological defects in silicene may be expected, maintaining however a peculiar stability even at high temperatures.

Remarkably, silicene and Si-NR exhibit, like graphene, massless relativistic Dirac fermions arising, for the nanoribbons case from the 1D projection of π and π* Dirac cones [26]. Moreover (see the relevant summary [27] and related references), the 1D topology which characterizes the metallic Si-NR structures favors the electrons interactions according with the *Luttinger liquid* model which implies the emergence of *bosonic quasi particles effects* coexisting with the Dirac fermionic characters expected for 2D silicene. On top of this, superconducting phenomena may be also expected for 1D silicene stripes matching similar effects measured at 8 K in hexagonal metallic silicon, possibly with an augmented T_c_ [26]. Van der Waals lattices of Group-IV elements made of germanenic (Ge-based) and stannenic (Sn-based) layers present analogous structural and topological features.

## 3. The Computational Method

The electronic properties of 1D nanoribbons are discussed here by considering the recent concept of bondon, the new bosonic quasi-particle arising from the Bohmian quantum picture applied to the quantization of the chemical bond [9,10,11,12,13,14,15,16] having the quantized proper mass:

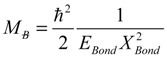
(1)

In Equation (1) the energy and the proper length of action obey to the Heisenberg analogous relationship [9]:

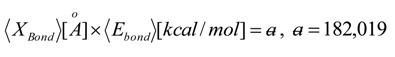
(2)

This description of the chemical bonding has been recently applied to extended nanostructures (*i*.*e*., for graphenic fragments sized in the range 15–30 Ȧ) with phase transformations [16]. Other applications includes the description of the optical and acoustic branches through the bondon-phonon interaction, the bondonic identification in the IR and Raman spectra of chemical compounds, as a measure of their reactivity or toxicity in bio-, eco- and pharmaco-logical cellular systems [28]. We describe in the following the phase transitions induced by the bondonic propagators till the 4th order (the maximum bond order in chemical systems) in Group-IV elemental defective nanoribbons.

One considers a particle (the bondon) with mass *M* moving between the space-points *x_a_* and *x_b_* under the potential *V(x*) to be further identified with the molecular net topological potential. The associate quantum evolution may be described by semiclassical propagator obeying the Schrödinger 1D equation, with the path integral solution being found in semiclassical expansion up to fourth (IV) order to look like (see Appendix 1) and [29,30]:

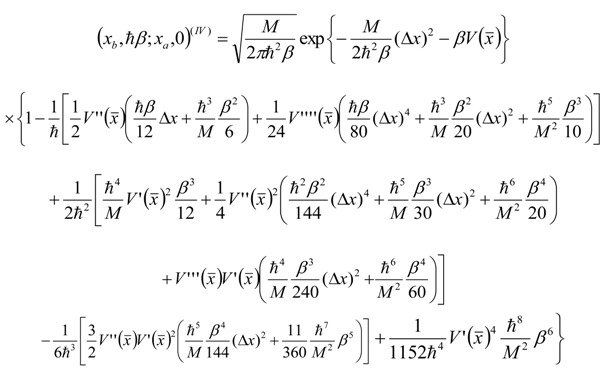
(3)

In terms of the classical path dependence connecting the end-points *x* = (*x_a_* + *x_b_*)/2 as well as on the path difference Δ*x* = *x_a_* - *x_b_*; here and throughout the whole paper *β* represents the inverse of the thermal energy *k_B_T* and *ħ* the reduced Planck constant. With Equation (3) one can form the partition function for the periodical quantum orbits by considering close integration over the classical or average path:

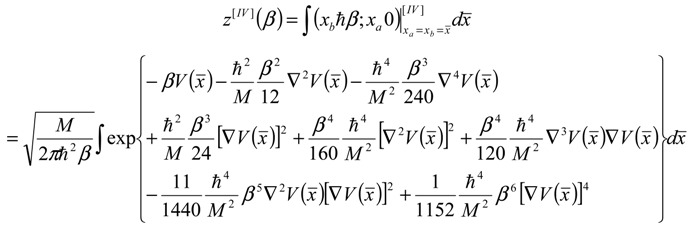
(4)

However, Equation (4) can be further simplified by using the Gauss theorem (see Appendix 2) to yield:

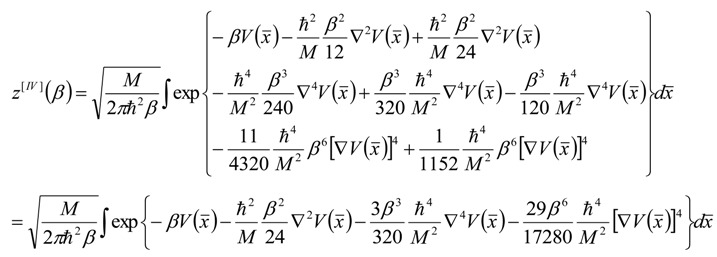
(5)

At this point one implements the bondonic information regarding the mass quantification in the valence state (the first or “ground” state of the bonding spectra) in terms of bonding energy and length of Equation (1), 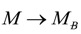, thus turning Equation (5) into the actual bonding related one:

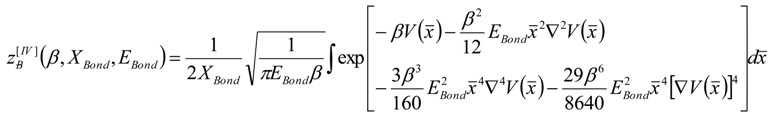
(6)

Now, facing the superior potential first, second, and fourth order derivatives, they can be systematically treated by replacing them with associate topological invariants and higher orders over the concerned bonds, networks or lattices, *i*.*e*.,:

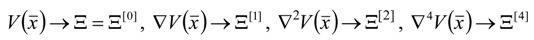
(7)

Nevertheless, attention should be paid at this passage from physical to topological quantities since it actually replaces *electronic interactions* with *topology-based interactions*, being therefore restricted to those topological invariants bearing an energetic meaning, as is the case with the Wiener index, for instance.

Next, one should fix the energy-length realm of the bondon in the 0^th^ order of the partition function which renders the classical observability by the involved thermal length, here mapped into the topological space and energy so defining the bondonic unitary cell of action [16]; to this aim, one firstly runs the 0th partition function:

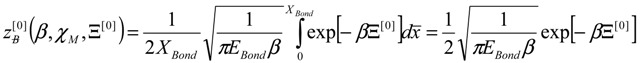
(8)

Then, Equation (8) is used for internal energy computing of the bondon as the average energy condensed in the network responsible for bonding at periodical-range action:

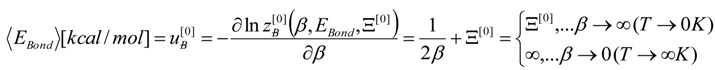
(9)

It immediately fixes the long-range length of periodic action of bondon by recalling Equation (2):

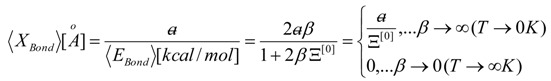
(10)

Remarkably, when the asymptotic limits are considered for both periodic energy and length of bondon, one sees that they naturally appear associated with the topological potential and with the Coulombian interaction for the low-temperature case, while rising and localizing the bonding information (like the delta-Dirac signal) for the high-temperature range, respectively, being the last case an observational manifestation of bondonic chemistry. This feature will be used in a moment below.

Returning to the full partition function now the bondonic periodicity information on length and energy action maybe included to rewrite Equation (6) to the actual form:

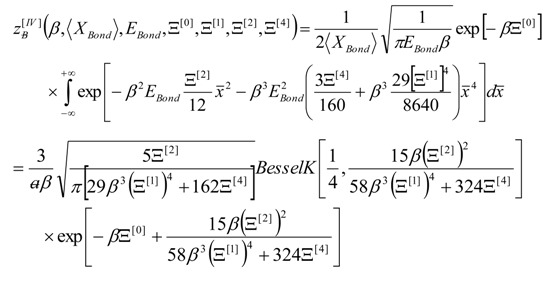
(11)

However, for workable measures of macroscopic observables, one employs the partition function of Equation (11) to compute the canonical associated partition function according with the custom statistical rule assuming the *N*-periodic cells in the network:

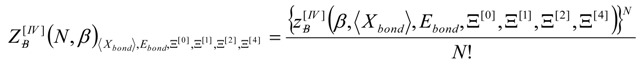
(12)

with the help of Equation (12) one is provided with the canonical (macroscopic) internal energy contributed by *N*-bondons from the *N* periodic cells, through considering further thermal derivation:

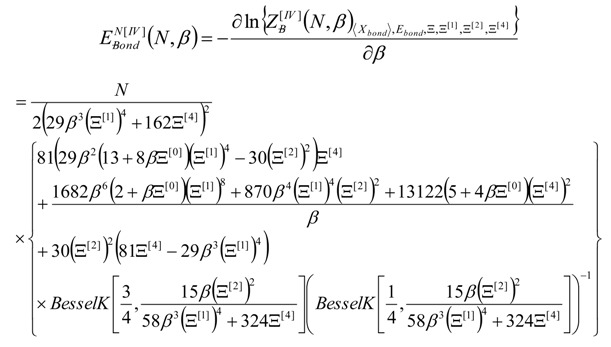
(13)

Finally; by continuing the inverse thermal energy derivatives; the internal energy of bonding of Equation (13) may be employed also for estimating the allied caloric capacity:

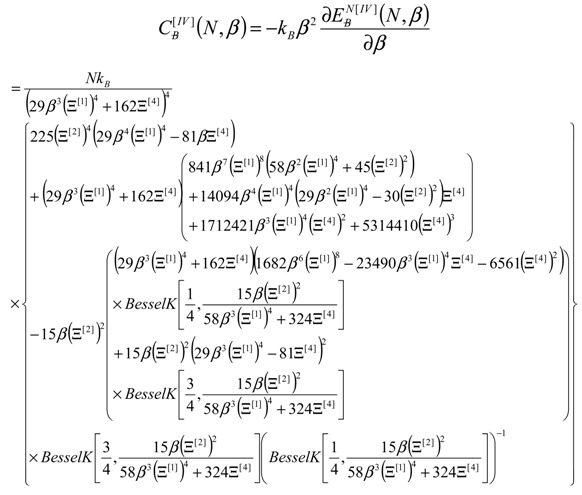
(14)

The treatment of pristine (“0”)–to–defect (“D”) networks goes now by equating the respective formed caloric capacities from Equation (14) towards searching for the *β*-critic through the phase-transition equation:

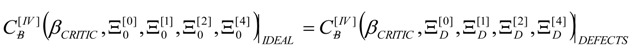
(15)

Now one may use the above mentioned high temperature regime, (*β* → 0), see Equations (9) and (10), in accordance with the present semiclassical approach, to find the critical phase-transition “temperature” to be:

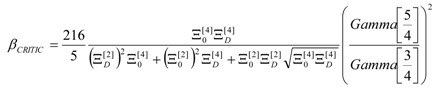
(16)

In the next section, this model will be applied to the study of the bondonic properties of graphenic (C-based), silicenic (Si-based), germanenic (Ge-based), and stannenic (Sn-based) nanoribbons with Stone-Wales defects.

## 4. Results and Discussion

### 4.1. Topological Wiener Polynomials

Here we will progress on the investigations of SW defects in graphene and related layers, as silicene germanene, and stannene, by analyzing the *propagation in the hexagonal nanoribbons* of the 5|7 pairs according to the wave-like topological mechanism originally introduced in [31] and called Stone-Wales waves (SWw) along with the effect a drifting effect may have on the long-range electronic properties of such monolayers with the aid of bondonic path integral formalism, just explained in a formal way.

**Figure 2:**
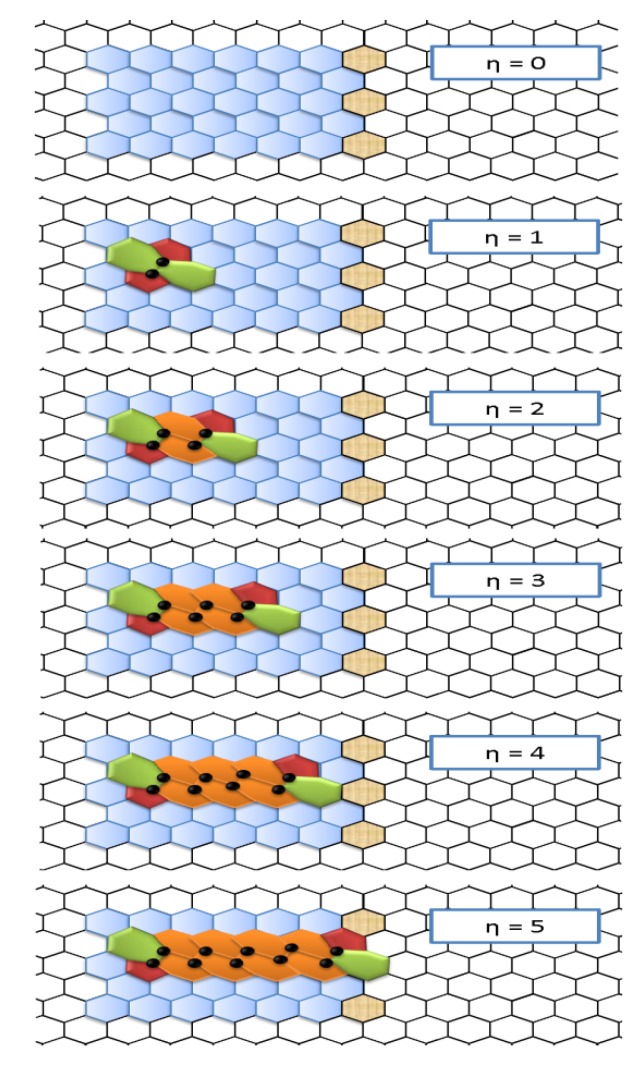
Propagation of the Stone-Wales wave-like defect along the zig-zag direction caused by the insertion of pairs of hexagons at *η* = 1, corresponding to the SW defect generations step; the size of this dislocation dipole ranges from *η* = 0 (pristine lattice) to *η* = 5; pristine (rearranged) hexagons are in blue (and orange); pentagons and heptagons are in red and green, respectively.

The topological skeletons of the systems considered in the present article are basically represented by a mesh of fused hexagons entirely paving the nanoribbons (Figure 2); closed boundary periodic conditions are imposed to form *nanotori* of carbon or silicon. Present section introduces to the graph-theoretical methods used to describe the *generation* and the *propagation* of the Stone-Wales defects in such a kind of *cubic* lattices, *i*.*e*., planar structures made of 3-connected nodes.

From the topological perspective, the key concepts applied in comparing graphene with silicone and related honey-comb networks’ properties are basically two:

- First, the two 5|7 pentagon-heptagon units SW constituting the SW defect, also called 5|7|7|5 dipole, are *considered free to migrate* in the hexagonal lattice by inserting *η*-1 pairs of hexagons 6|6.

Such a structural modification reflects *a universal topological property of the hexagonal meshes*. In this way, extended linear defects are created, keeping the modified structures *fully isomeric* to the initial one (*i*.*e*., conserving number of atoms, rings and bonds). Figure 2 shows, from the graphical point of view, the iterative sequence of bond rotations producing the *SW dislocation dipole* 5|7{6|6}*_η_*7|5 with size *η* also called *SW wave* (SWw) [31]. DFT computations [32] demonstrate that strain-induced local forces energetically favor the topological swap of two hexagons with the pentagon-heptagon pair; more details on topological dislocations are provided in the original paper on SWw [31]. Although SWw defective configurations are not yet investigated in Si-NR systems by mean of *ab-initio* methods, the introduction-mentioned *E_b_* ≈ 2.8 eV low values featured by the energy-barrier for normal SW rotations in Si hexagonal layers (see reference [27]) encourage the search for that defect diffusion mechanism also in silicene. 

The second conceptual instrument used in the present analysis regards the physical-to-topological passage introduced by Equation (7):

- The evolution of the nanoribbon defective structure is controlled by a pure *topological potentials Ξ* expressing the long-range, collective effects of the network on the network stability itself in terms of distance-based topological invariants computed on the nanoribbon chemical graph composed by *n* nodes.

Equally important, *topological potentials* *Ξ* are subject to a minimization principle. In spite of this apparently simple statement, appropriate approximation demonstrates in several cases a substantial predictive power when the *topological potentials* *Ξ* are applied for studying the *isomeric* evolution of *complex systems*, like the *SWw-surfed nanoribbons* under present investigation. An overview, from “fullerene to graphene”, of *topological modeling* simulations is provided in [33], whereas article [16] presents the first investigation of the influence of the collective topological properties of honeycomb lattices over the collective bosonic behavior of *sp*^2^ electrons. 

It is worth remembering here the “basal properties of distance-based topological potentials“ making those mathematical object exceptionally suitable for determining delocalized bondonic properties:

(i) *physically*, the topological potential *Ξ* considers *by definition* the collective long-range effects produced by the mutual interactions of all atoms pairs of the chemical system;
(ii) *numerically*, *Ξ* features an easily-manageable *polynomial behavior* in term of the parameter expressing the *size of the system* (that parameter may be *n* or even *η*) with the leading coefficient of the respective polynomial only depending *from the dimensionality* D of the system–see the recent review on topological modeling methods and results [33].

Actually, a practical introduction to lattice topological descriptors is provided by looking to the nanoribbon structure in Figure 2 as an hexagonal network with *n* atoms. While indicating with *d_ij_* the *ij*-element of the *n* × *n* distance matrix *D* of the graph, the first important lattice descriptor is represented by the topological Wiener index *W*, e.g., the semi sum of the *n*^2^ entries of:

*W* = ∑_*i*>*j*_* d_ij_ with d_ij_* = *0* (17)

The invariant Equation (17) provides a powerful rank of isomeric chemical graphs, privileging the most compact structures [33]; for this reason, the Wiener index is a natural choice for the role of chemical potential of the system, continuing Equation (7) here with the involvement of the energetic calibration slope (*α*):

*Ξ ^W^*=*αW* (18)

Systems like graphene and, to some extent, the related ones, including silicene, that are rich in *sp*^2^ electrons, are conveniently described by introducing an explicit term in the electronic potential energy to convey the effects of *conjugation forces* among the occupied states of the unfilled *π*-bands. As recently demonstrated in [34], that electronic *conjugation* term involves the lattice topology, being *directly* proportional to a combination of the Wiener index *W* Equation (17) and the order *s* corrections:

*W^(s)^* = ∑_*i>j *_*d^s^_ij_ with d_ii_* = *0* (19)

In case of large structures only the first terms *s* = 1,2,3,4… contribute significantly to the global energy, and proper scale factors *γ_s_* have to be computed in the relative expression for the topological potential:

*Ξ ^W^* = *∑_s_ γ_s_W^(s)^* with *s* = 1,2,3,4… (20)

Interested readers may find the formal derivation of Equation (19) contributions and related asymptotic properties in the original work [34]. For *s* = 1 (this is the case of large lattices) Equation (20) reduces to Equation (17) with *γ*_1_ being the energy scale factor one may interpolate by *ab-initio* results. Topological invariants *Ξ* are computed for the defective isomeric configurations illustrated in Figure 2. The nanoribbon building unit is made of *n*_0_=84 atoms constituting the colored rings. In order to avoid long-range self-interactions, topological potential are computed in a *periodically closed* supercell *E* built by (3 × 3) building units. Supercell *E* has therefore a grand-total of *N =* 756 nodes and *B* = 1,134 chemical bonds (or graph edges), the *B* = 3*n*/2 relation being valid for other cubic graphs like the fullerene ones. At the center of that supercell, the *n*_0_ = 84 array will hosts the generation and the propagation of the *η*-sized Stone-Wales wave for *η* = 0,1,2,3,4,5 the *η* = 1 step corresponding to the generations of the standard SW defect 5|7|7|5. In Figure 2 the black-circled atoms mark the bonds rotated during the expansion of the SWw dislocation dipole. For the nanoribbon fragments of Figure 2, through employing the pristine-to-defective steps *η* = 0-5, the topological potentials need in Equation (7) are generated by the associate polynomials of Equations (21)–(24), respectively:

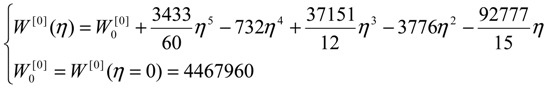
(21)

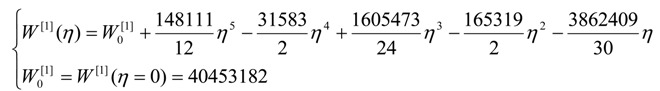
(22)

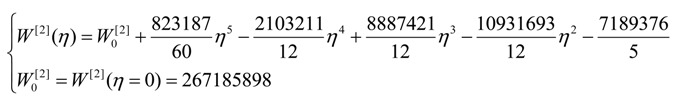
(23)

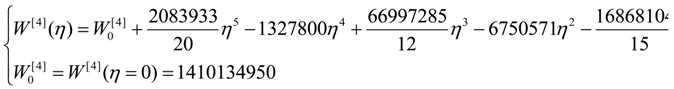
(24)
with the specialization for each defective nanoribbon-steps depicted in the Figure 2 and reported in Table 1.

**Table 1:** Numerical values abstracted from topological potentials of Equations (21)–(24) then used to generate the interpolations polynomials of Equations (27)–(34) as a function of the *η*-step of the forming (*η* = 0,0.2,0.4,0.6,0.8,1) and propagation (*η* = 0,1,2,3,4,5) of the SWw dipole in the periodic nanoribbon supercell ***E*** of the of Figure 2, respectively, see text.

η	W^[^°^]^	W^[1]^	W^[2]^	W^[4]^
0	4,467,960	40,453,200	267,186,000	1,410,130,000
0.2	4,466,600	40,424,600	266,868,000	1,407,660,000
0.4	4,465,060	40,392,500	266,508,000	1,404,880,000
0.6	4,463,470	40,359,500	266,134,000	1,402,000,000
0.8	4,461,900	40,329,100	265,764,000	1,399,170,000
1	4,460,420	40,305,200	265,416,000	1,396,500,000
2	4,455,370	40,542,500	264,226,000	1,387,400,000
3	4,453,620	42,849,300	263,807,000	1,384,160,000
4	4,452,140	51,493,100	263,439,000	1,381,240,000
5	4,450,930	74,805,700	263,132,000	1,378,770,000

The supercell in Figure 2 shows *two distinct topological regimes* according to the selected topological potential. Considering *Ξ ^W^* = *αW* as the potential energy of the system, see Equation (18) and Table 2, the generation and the propagation of the isomeric SWw dipole results in a topologically favored condition. The system evolves in such a way the Wiener index Equation (17) decreases with *η* = 1 by reducing the chemical distances in the graph in the 7-rings region. Only the *W*^[1]^ presents an anomaly to this behavior as illustrated in Table 1, starting for the steps *η* = 4&5; this justifies the present fourth high order approach in order to properly describe complex topological electronic bonding features as well, within the frame of the bondonic formalism. Nevertheless, other terms in the topological potential, coming from W^[2&4]^ descriptors, follow the *W*^[0]^ behavior and they do not alter therefore this compactness-driven propagation effect along the zig-zag edge of the nanoribbons; numerically, topological distances span the *d_ij_* = 1,2,…,29,30 range in all the lattice configurations with *N =* 756 nodes whose central defective regions (having *n*_0_ = 84 atoms) are step-by-step reproduced in Figure 2.

**Table 2:** Synopsis of the topo-reactive parameters for the defects instances (starting from pristine net at the step *η =* 0) from the SW propagations in Graphene sheet as described in Figure 2, namely: electronic, total, binding and parabolic energy–the last one computed upon Equations (25) and/or (26) with the total number of pi-electrons N_π_ = 82 for the steps *η =* 0÷4 and N_π_ = 84 for the last instant case *η =* 5, within the semi-empirical AM1 framework [39], respectively; the bottom of the table reports the free intercept correlation slopes and the associate correlation factors for each set of structural energies respecting the topological defective Wiener potential values of Table 1, providing the actual hierarchy (bolded values) and the calibration recipe (bolded italic) then used to generate the working potential polynomials of Equations (27)–(34).

Defect Step	Instant Structure	Electronic Energy (eV)	Total Energy (eV)	Binding Energy (eV)	Parabolic Energy (eV)
*η = 0*	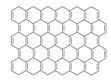	2858.69979	2595.306	7308.17	13,063.1207
*η = 1*	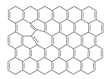	2425.90314	2409.47	7494.0069	102,344.109
*η = 2*	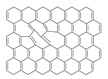	2641.35644	2410.023	7493.4534	100,091.3384
*η = 3*	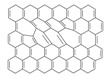	90063.404	10331.43	−427.9522	6770.427006
*η = 4*	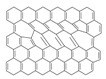	2428.23769	2408.129	7495.3472	102,353.338
*η = 5*	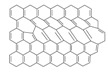	2484.84517	2468.133	7676.8912	107,394.1399
*Correlation Slope, α in* Equation (18)	*W*^[0]^(*η*)	0.00384593	0.000846	**0.0013853**	0.01614977
	*W*^[1]^(*η*)	0.00029961	7.01 × 10^−5^	**0.000123**	0.001488221
	*W*^[2]^(*η*)	6.4681 × 10^−5^	1.42 × 10^−5^	**2.335** × **10^−5^**	0.000271766
	*W*^[4]^(*η*)	1.2299 × 10^−5^	2.71 × 10^−6^	**4.445** × **10^−6^**	5.16926 × 10^−5^
*Correlation Factor R^2^*	*W*^[0]^(*η*)	0.21643315	0.622413	**0.813889**	0.727612957
	*W*^[1]^(*η*)	0.16519269	0.538169	**0.8067669**	0.777077187
	*W*^[2]^(*η*)	0.21568411	0.621567	**0.8144879**	0.725935217
	*W*^[4]^(*η*)	0.21522938	0.621048	**0.8148337**	0.724864925

### 4.2. Topo-Reactivity Wiener Polynomials

The appropriate *α* values in Equation (18) are determined by a specific interpolation process that is described in the following. To model chemical reactivity, one considers various energetic quantities (such as the total energy, electronic energy or binding energy) alongside the celebrated parabolic form of the pi-energy [35,36] computed by mean of a polynomial combination of *electronegativity* and *chemical hardness* for frontier orbitals (such as HOMO-highest occupied molecular orbital and LUMO-lowest unoccupied molecular orbital) respecting the number of pi-electrons engaged in the molecular reactivity; as such it runs upon the Mulliken-type formula [37]:

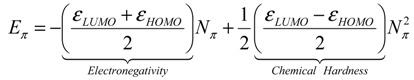
(25)

Equivalently, within the frozen core approximation or by Koopmans’ theorem [38], it is rewritable in terms of the ionization potential (IP) and electronic affinity (EA):

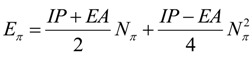
(26)

Accordingly, Table 2 displays [39], respecting the defect-step evolution, the numerical values of these energies during the propagation of SWw defects in graphenic nanoribbons. The “best” free intercept correlation, as in Equation (18), with the corresponding series of Wiener topological indices of Table 1 is then computed for each energetic frameworks considered in Table 2, deriving the related correlation factor hierarchy. One notes that, in line with above observations, only the correlation output in the step *η* = 1 is systematically spurious in respect to the remaining correlation factors, most probably due to the dispersive effect present in the first order derivatives of the topological potential, the same dispersive effect being also present in the physical picture of dissipation phenomena [40]. 

Also interesting, the parabolic based chemical reactivity analysis furnishes the second best results after the pure binding energy correlations; this behavior justifies both the pro and contra regarding its use in modern chemical reactivity theory, namely:

- the pro-argument, largely advocated by Parr works in last decades of conceptual chemistry research with application in inorganic and organic reactivity alike [41,42,43,44], while being recently employed by present authors in “coloring” chemical topology with chemical reactivity electronic frontier information of atoms in molecules
- the contra-argument, defended by late Szentpaly works on various inorganic systems [46], according which the parabolic description is slightly non-realistic neither for ground nor for valence state of atoms and molecules since actually not having the minimum of the parabola on the right realm of exchanged electrons in bonding or in ionization-affinity processed; this limitation was also conceptually discussed by one of the present authors in a recent paper advancing the cubic form of chemical reactivity as a better framework for conceptual treatment for electronic exchange as driven by electronegativity and chemical hardness, with an universal (and also Bohmian) value [47].

Therefore, although valuable, the parabolic reactivity calibration is also by this approach taken over by the cute binding energy for the correlation coefficients with topological potentials in Table 2, even at semi-empirical level — nevertheless in the line with the present semiclassical methodology. Once the correlation framework was established for according the topological with energetically passage of Equation (18) at its turn completing the recipe of Equation (7), one may further interpolate the creation and propagation of the SWw in the honey-comb nanoribbons of Figure 2 by employing the data of Table 1 and then appropriately calibrating the fifth order polynomials for the two cases, respectively:

- the energetically calibrated topological potentials for the forming SW defect instance (still corresponding to the “0” structure) within [0-1] range of the *η* “steps”:

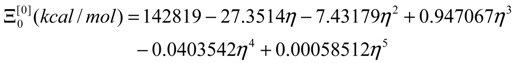
(27)

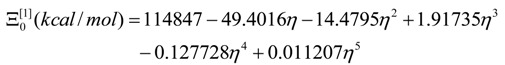
(28)

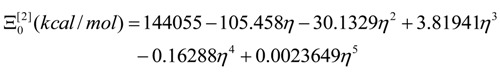
(29)

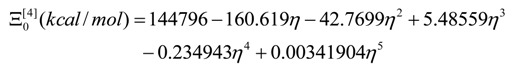
(30)
- The polynomials for the topological potentials describing the SW waves still corresponding to the defective “D” structures) within [0-5] range of the *;* steps

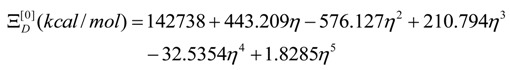
(31)

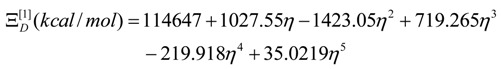
(32)

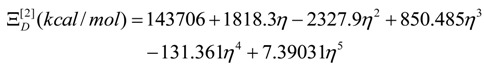
(33)

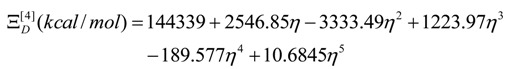
(34)

It is worth evidencing the advantage of this procedure which effectively allows an easy energetic calibration and a separate description of the 0-forming and D-propagating steps of the SWw defect by providing the associate polynomials that are: (i) energetically realistic and (ii) with “equal importance” despite the different information contained: see for instance the numeric form of the fourth order topological potential Equation (24) respecting those provided by Equations (30) and (34). This computationally-convenient method assures that higher order topological potentials will contribute in providing the bondonic related quantities of Equations (13), (14) and (16).

### 4.3. Bondonic Effects on Topological Defects in Group IV-Honeycomb Nanoribbons

Nevertheless, all the present computational algorithms were implemented for graphenic structures, having the carbon atom as the basic motive; however they can be for further used in predicting similar properties also for similar atomic group like Si, Ge, Sn, through appropriate topological potential factorization depending on the displayed reactivity differences; since such differences are usually reflected in gap band or bonding distance differences, one may recall again the electronegativity as the atomic measure marking the passage from an atomic motive to another keeping the honeycomb structure. The influence of the lattice will be implemented by considering the (electronegativity dependant) function of the fermionic statistical type with 2-degeneracy of states spread over the graphenic type lattice–taken as a reference. Such a function accounts for the electronic pairing in chemical bonding is analytically taken as:

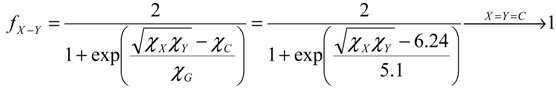
(35)

Numerically, Equation (35) features the factorization with unity for C-C bonding, while departing to fractions from it when the Group A-IV of elements are considered as motives for honey-comb lattices with graphenic reference: Si-Si honey-comb bonding will carry statistically the Si atomic electronegativity χ(Si) = 4.68 [eV] in Equation (35) with X = Y = Si, and successively for Ge-Ge with χ(Ge) = 4.59 [eV], and Sn-Sn with χ(Sn) = 4.26 [eV] for the corresponding silicone as well as for similarly designed germanene and stannene nanoribbon structures. Note that atomic electronegativity were considered within the Mulliken type formulation of ionization potential and electronic affinity as in the first term of Equation (26); furthermore, their geometric mean was “measured” against the referential graphenic C-C chemical bonding, while their difference was normalized under exponential of Equation (35) to the so-called “universal” geometrical averaged form of Parr and Bartolotti, *χ_G_* = 5.1 [eV], at its turn obtained within the electronegativity geometric equalization framework [48]. Note that the present approach may allow for further extension towards XY hetero-bonding arranged in honey-comb lattice in which cases the mixed combinations C-Si, C-Ge, C-Sn, Si-Ge, Si-Sn, and Ge-Sn are implemented following the same formalism. Yet, here we will be restricted to homo-bonding in nanoribbons only due to their specific van der Waals interaction. 

Going to have the final and most important part of discussion of the obtained bondonic observable properties through the present fourth order topological-potential formalism, they will be displayed through jointly implementing the short and medium (*η* = 0–7) to long (*η* = 0–10…50) range effect of the present interpolation-calibrated potentials Equations (27)–(34); in other words, although having obtained the working topological potentials over a finite “movie” of forming and propagating SW defects in Figure 2, by letting “free” the step argument η in the actual evaluated quantities of Equations (13), (14) and (16) one actually will explore how much the short range behavior will echo into the long range as well, or whether this echo will feature some distortions, peaks or valleys, equivalently with a predicted signal to be recorded on extended nanosystems of graphenic type.

**Figure 3:**
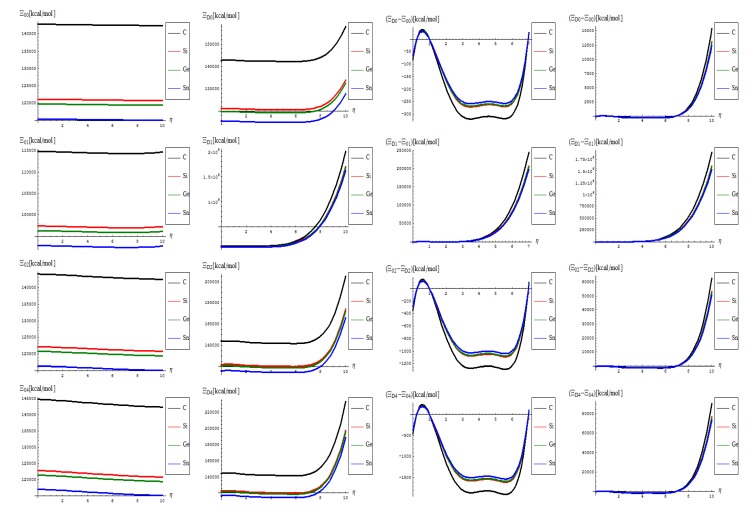
The Wiener based topological potentials of Equations (27)–(34): from top to bottom in successive orders and from left to right for the forming (“0”) SW and for propagating (“D”) of the SW defects on the medium (*η* = 0–7) and long range (*η* = 0–10) range, respectively.

One starts with the topo-energetic Wiener potentials of Equations (27)–(34) with representations in Figure 3 noting that:

- The topological potentials modeling the forming (“0-to-1”) step of SWw are all monotonically descending, meaning their eventual release into defective structures;
- The topological defective potentials have quite constant behavior over the entire computational *η* = 0-5 plateau, while recording quasi-critical rise for the potential “echo” spanning the long range behavior, meaning the rising of the defective potential barrier in fact (with more emphasize for the first order potential, as expected from previous discussion, see Table 1, for instance); this strongly suggest the real finite range for the defective SWw, *i*.*e*., the annihilation long-range stage that came after their creation on the short-range realm;
- The D-to-0 difference shows some short range fluctuations for all potentials unless the first order one, yet ending into the D-potential definite rising barrier on the long range behavior; the difference for the first order potential displays such energetic barrier rise just from the short range, paralleling the defective “D” shape;
- Concerning the C > Si > Ge > Sn potential (paralleling the electronegativity) hierarchy, one sees that the C-to-Si large energetic gap for pristine “0” structure is considerably attenuated for the “D” propagation of the SWw defect, manifested especially for second and 4th order, while the Si-to-Ge energetic curves almost coincides for these orders; even more, all Si, Ge, and Sn shapes are practically united under first order defective potential “D1”.

**Figure 4:**
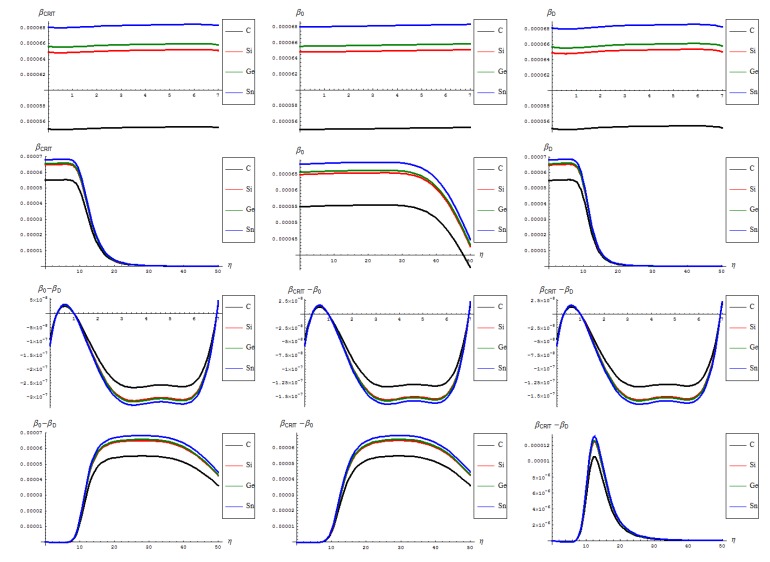
The critical (left column), forming (middle column) and transforming (right column) of SWw in Figure 2, as based on Equation (16) on the short (first upper row) and long range (second upper row), and of their respective differences.

These topological potential features stay at the foreground for further undertaking of the remaining observable properties in a comparative analysis framework. As such, when analyzing the critical “temperature” through the inverse of the thermal energy of Equation (16) one actually gets information on the phase transition *specific time* (via the celebrated statistical-to quantum mechanics equivalence facilitated by the Wick rotation, *ћβ*↔*τ_β_*) for SW forming, propagating and disappearing through the isomeric nanoribbons of Figure 2, with the dynamic representations of the Figure 4:

- The general feature for the *β* signals is that it is constantly for the short range in phase transition (critical curve) between forming (“0-to-1”) and transforming “D” of SW waves and along the predicted inverse C < Si < Ge < Sn signal (pulse) hierarchy;
- The situation changes on the long range “echo” when the critical signal may be recorded closer to the defective than to the pristine structures, when one should record also a shrink signal pulses gap between the C-to-Si-to-Ge-to-Sn;
- The differences between the critical-to-defective-to-pristine structures’ pulses shapes follows on a short range the generally recorded topological potential difference in that range, while noticing definite cupolas for the long range behavior–especially on the critical regime, meaning that indeed the SWw echo is disappearing after about 50 isomeric topological transformation of the considered honey-comb nanosystem (see the right bottom line picture of Figure 4).

It is worth noting that the *β* signals of Figure 4, when measured in seconds, through the [kcal/mol]-to-[Hz] transformations [49] since the Equation (16) relationship with topological potentials expressed in [kcal/mol] surpass the current femtosecond limit (attainable only by synchrotron measurements); however, one can equally asses that these shorter times are specific to isomerisations or topological rearrangement processes; they are however no more “theoretical” having from the present study an associated scale and prediction algorithm. It is equally possible to imagine other nanosystems for which *β* has higher and therefore shorter times of detection for topological isomers.

**Figure 5:**
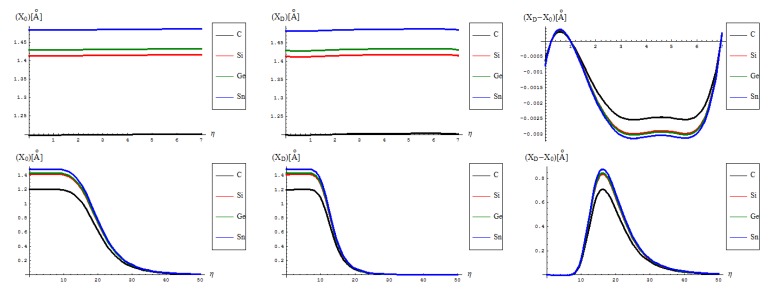
The bondonic “length” for SWw in forming (left column), defective propagation (middle column) along their differences (right column) behavior: on short (upper row) and long (lower row) ranges, upon considering critical information of Equation (16) into Equation (10).

Even more, these times are in fact the bondonic times for concerned lattices whose periodic radii of action is determined upon considering the *β* information in the specific Equation (10) featuring the Figure 5 representations and the following characteristics:

- The identical boning length for pristine and defective structures on the short range transformations (up to seven topological rearrangements paralleling the SW wave propagation into extended lattice); notably, the bondonic lengths are correctly shorter than the detected or previously estimated bonding length for C-C bond (in graphene) and elongated in Si-Si bond (in silicene), see the Introduction, since the bondonic agent nature, in assuring the bonding action for the basic atomic pairing in honey-comb nanoribbons.
- The decrease of boning length and of the consequent action on the long range dynamics, paralleling the decreasing of the inter-elongation difference in bonding for C-to-Si-to-Ge-to-Sn;
- The prediction on the bondonic longest “echo” in an extended lattice, limited to 50 transformations, or dipole extensions steps continuing the Figure 2; this information has a practical consequence in predicting the longest nano-fragment still chemically stabilized by the bondonic “echo” by its long range action (sustained by its inner Bohmian nature, see reference [9]);
- The D-to-0 bondonic length differences parallels those recorded for the critical-to-D one found for the *β* signal in Figure 4, this way confirming the finite (non-zero nor infinite) physical length over which the phase transition from pristine to Stone-Wales topological isomer is taking place; it also offers an spatial alternative to temporal (quasi-inaccessible) scale of measuring and detecting the bondonic effects on topological transformations and isomeric rearrangements.

**Figure 6:**
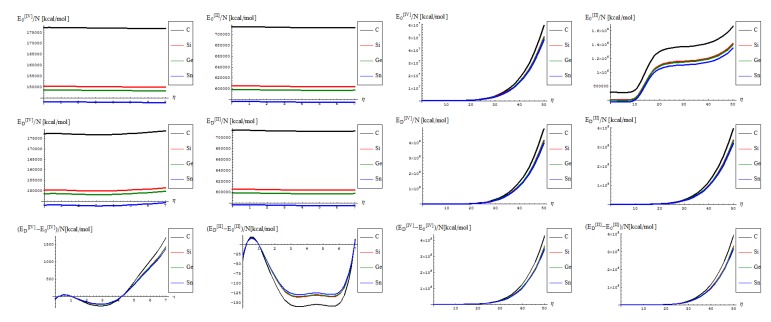
Side-by-side canonical internal energies of bondons in honey-comb supercells of Figure 2, as computed with the fourth order formulation of Equation (13) side by side with the former second order formulation of reference [16], for pristine “0” (upper row), defective “D” (middle row) and their differences (lower row), respectively.

Passing to the canonical measures one has in Figure 6 the representations of the sample total energy representations in “0” and “D” sates, and their behavioral difference, side by side for the actual fourth order algorithm with the previous second order restricted treatment, see reference [16]. Accordingly, the specificities can be listed as follows:

- No shape differences other than the overall scales along a quasi invariant energy-gap between C-to-Si-to-Ge-to-Sn related lattices are recorded between the [II] and [IV] order path-integral bondonic formalisms, with natural higher energetic records for the later approach since more interaction/interconnection effects are included, for the internal energies of honeycomb lattice without and with topological defects as triggered on the short range as in Figure 2;
- The previous situation changes for the long range SW dipole transformations, noticing the same type of energetic increase as for the forth order topological potential/barrier in Figure 3, together with quasi-unifying the C with Si-to-Ge-to-Sn behaviors (being the last three atomic based lattices quite unified in total bondonic energetic shape); the peculiar behavior is noted just to [II] order treatment and in the long range pristine super cell self-arranging, when the energy rising displays two plateaus as well as still a C respecting Si-to-Ge-to-Sn energetic gaps for their honeycomb lattices; however, the energetic rising even for the so called pristine structures is in accordance with the bohemian quantum nature of the bondon which accounts for the self-arrangements of a quantum structure even when self-symmetric, being this in accordance with quantum vacuum energy which is non-zero due to the energy required for internal self-symmetry eventually broken in the spring isomerisations of space, here represented by the SW topological defects and of their (also finite) propagations.
- The D-to-0 differences are nevertheless replicating the defective behavior for both the [II] and [IV] order analysis on the long range, while showcasing some different types of fluctuations and inversions along the C-to-Si-to-Ge-to-Sn honeycomb nanosystems for the short-range of SW dipole evolution;
- with special reference to [IV] short range behavior one notes the the pristine “0” state is still present as an “echo” over the defective “D” state, due to its energetic dominance, that nevertheless has the contribution in replicating “the learning” mechanism of generating SW defects in between each short range steps as was the case in between η = 0 and η = 1; remarkably, this may have future exciting consequences in better understanding the cellular morphogenesis by “replicating the learning” machinery of the Stone-Wales transformation, found to be present also at the cell-life-cycle phenomenology, see [28].

**Figure 7:**
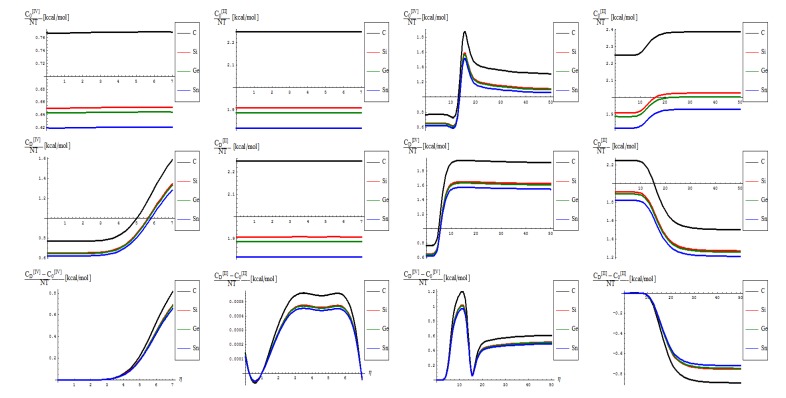
The same type of representations as in Figure 6, here for caloric capacity of Equation (14) and of former formulation of reference [16], in the fourth and second order path integral of bondonic movement, respectively.

Going to the last but the most “observable” quantity which is the caloric capacity of Equation (14) within the present [IV] order path integral–bondonic approach, one has the results, and comparison with the previous [II] order formalism of reference [16], exposed in Figure 7, with the notable characteristics that follow:

- from the scale values, one obtains actual quite impressive accordance with the previously calculated or predicted values for the graphene and silicone networks: take for instance just the pristine “0” output, in [IV] other environment; for it one notes the constant results about 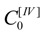/(*NT*)[*kcal*/*mol*] ~ 0.77 for graphene and 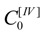/(*NT*)[*kcal*/*mol*] ~ 0.65 for silicene; when taking account of the units transformations [49] such as 1 [kcal/mol] = 503.228 [K], one arrives that, for instance, for room temperature of T~300 K, and for short range transformation (say *N =* 7 bondons involved, one created per each step of topological transformation) one gets 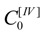
(*C*)[*hartree*] ~ 0.185 and respectively 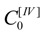
(*Si*)[*hartree*] ~ 0.156, which in [eV] will respectively give about 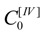
(*C*)[*eV*] ~ 5.09 and 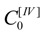
(*Si*)[*eV*] ~ 4.24 which nevertheless are quite close with previous estimations for SW rotation barriers as *E_b_* ≈ 5 eV for graphene and *E_b_* ≈ 2.8 eV for silicene (see Introduction); the discrepancy may be nevertheless avoided while considering the semiconductor properties of Si which requires more bondons being involved such that the SW rotational barrier to be passed and the defective dipole triggered; as such for *N =* 10 created bondons for Si–SW super cell one refines the above result to 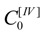
(*Si,N* = 10)[*eV*] ~ 2.96 which fits quite well with literature results, see reference [25]. On the other hand, it is also apparent that for [II] order treatment the data of Figure 7 implies that more bondons are required to fit with the right observed or by other means estimated data, which leaves with the important conceptual lesson: more bondons–less connectivity relationship, very useful in addressing other fundamental chemical problems like crystal field theory and aromatic compounds, just to name a few.
- As previously noted the “D” effect is to shrink the energetic gap between C-to-Si-to-Ge-to-Sn lattice structural behavior, respecting the “0” pristine or defect forming transition state;
- The short range D-to-O differences closely follow the previous internal energy shapes of Figure 6, yet with less oscillations for the [IV] treatment, thus in accordance with more observable character of the caloric capacity;
- For the long range behavior, instead, what was previously a parabolic increase in internal energy acquires now a plateau behavior in all [IV] and [II] order representations: “0”, “D”, and their “D-to-0” differences; nevertheless it seems that the “echo”/signal about η = 10 is particularly strong in [IV] order modeling of D-to-0 differences in caloric capacities, while we notice for the “D” state the graphenic apex curvature about η = 7 followed by that of silicone at η = 10, in full consistency with above bondonic energetic analysis (*N* = 7 for C-lattice, and *N* = 10 for Si lattice), thus confirming it. Further signals are also visible, for accumulation of bondons (as the SW dipole evolve and extends over the nanostructure) at η = 15 in pristine structure, as well as for the further plateaus within the [II] order analysis, again in accordance with the above discovered rule of more bondons being required for acquiring the same effect with less connectivity (long-range-bonding neighboring) analysis.

The present results fully validate bondonic analysis as a viable tool for producing reliable observable characters, while modeling and predicting the complex, and subtle, chemical phenomenology of bonding in isomers and topological transformations in the space of chemical resonances. Further works are therefore called in applying the present algorithm and bondonic treatment for other nanosystems as well as in deep treatment for the symmetry-breaking in chemical bonding formation of atoms-encountering in molecules and in large nanosystems.

## 5. Conclusions

The current article aims to contribute in advancing the fascinating *quantum topological theory* by treating the 1D hexagonal meshes (nanoribbons) with original studies of peculiar topological, large-scale defects and quantitative predictions for the collective bosonic-like electronic behaviors for C, Si, Ge and Sn systems, while paving the way also for further hetero and alloy nanosystems, honoring Novoselov’s Nobel Lecture who invokes for all the magic one may (hopefully) encounter in Flatlandia [50].

Highly intriguing novel properties are theoretically derived here, namely: the topological potentials up to the fourth order, the so called beta-signal accounting for the time scale of bondonic pulses in a lattice supercell of graphenic type, the associate length of action, along the total internal energy of topological isomers and the remarkable behavior of the caloric capacity of the nanosystems. These quantities were evaluated and discussed for a critical regime by modeling the phase transition from pristine to defective nanoribbons with Stone-Wales dislocation dipoles and the creation of isomeric bondons; anti-bonding particle creation have been also described.

As an overall comment, the ability to indicate peculiar scale-threshold (like the *η* scale) for a given process in nanosystems, represents a relevant computational result which provides an increasing importance to topological simulation algorithms. *Ab-initio* models hardly compete with topological modeling in selecting/proposing interesting configurations in extended nanosystems involving hundreds or thousands of atoms like the structures studied here; they have nevertheless a key role of refining the physical characterization of those proposed configurations, assessing their physical-chemical relevance. The typical example is the Stone-Wales wave isomeric mechanism that produces *η*-extended dislocation dipoles in graphene-to-silicene nanoribbons; future computational studies, especially at DFT level, will be necessary to describe and cross-check the actual bondonic findings regarding the energetic barriers and thermodynamic stability of SW topological defects in Group-IV elemental honeycomb and related hetero structures.

Nevertheless, future applications triggered by the present study of graphenic systems are extendible to the design of reactive supports for pharmaceutical and cosmetic compounds, due to their unique electronic, magnetic and chemical saturation properties (recognized by the Nobel Prize in Physics in 2010), while the technological passage downward Group-IV elements is expected to enrich the *moletronics field* with semi-conductor and quantum electronic exotic properties.

Finally, the bondonic quantum condensate distribution picture allows the computation of the energetic (observable) energies involved in the isomeric nanostructures with the exciting perspective of simulating the creation and dissipation of SWw defects in the graphenic-like regions characterizing the surface of *large* fullerenes, by modeling at the same time the creation and the annihilation of bondons. So far, defective fullerenes modified by sequences of isomeric SWw topological transformations are totally unexplored and their bondonic modeling will be soon investigated. The isomerisation of honeycomb nanostructures by the application the generalized Stone-Wales transformations formalism is fully extendable to the case of non-spiral fullerenes as demonstrated by the recent study [51].

## Appendix

### Appendix 1: Derivation of the 4th Order Path Integral Based Quantum Propagator

Path integrals quantum formalism represents the viable integral alternative to the differential orbital approaches of many-electronic systems at nanoscale; accordingly, by employing path integral formalism one actually avoids the cumbersome computation and modeling of the orbital wave-function. Current method is therefore best suited for the path integral approach to the extended systems in which topological and bondonic chemistry appropriately describe long-range structures and the long-range interactions, respectively.

Accordingly, one looks for evaluating the space-time quantum amplitude/propagator (*x_b_*,*τ_b_*|*x_a_*,*τ_a_*) rather than the wave-function or electronic density for a characteristic potential of a given system. The quantum propagator can be analytically expressed by considering the parameterized quantum paths *x*(*τ*) = *x* + *η*(*τ*) where the classical path is replaced by the fixed (non time-dependent) average *x* = (*x_a_* + *x_b_*)/2, while the fluctuation path *η*(*τ*) remains and accounts for the whole path integral information to the actual different endpoint values *η_a_* = *η*(*τ_a_*) = -Δ*x*/2, *η_b_* = *η*(*τ_b_*) = +Δ*x*/2; the working quantum statistical path integral representation of the time evolution amplitude has therefore the input form [52]:

(A1)

that turns into its semiclassical form upon the potential expansion respecting the path fluctuations, here up to the fourth order contributions:

(A2)

Note that we have maintained here the physical constants appearances so that the semiclassical expansion procedure is clearly understood in orders of Planck’s orders, see below. As such, the path integral propagator representation can be summarized as:

(A3)

by introducing the so called *free* imaginary time amplitude:

(A4)

readily given by the free-propagator solution [53]:

(A5)

while having the normalization role for averaging the semiclassical factor contribution:

(A6)

All in all, the semiclassical form of path integral representation of evolution amplitude looks in the fourth order of Planck constant’s expansion:

(A7)

Now, we are faced with expressing the averaged values of the fluctuation paths in single or multiple time connection, *i*.*e*., ˂*η_i_*(*τ*)˃, ˂*η_i_*(*τ*)*η_i_*(*τ*)˃, ˂*η_i_*(*τ*)*η_i_*(*τ'*)˃, *etc*. For that, it can be readily shown that the first order of the averaged fluctuation path resembles the classical (observed) path:

(A8)

while the higher orders can be unfolded in connection with the Green functions/propagators according to the so called *cluster decomposition* or *cumulant expansion*:

(A9)

However, by involving the pair-wise (Wick) decomposition of the *n*-points correlated function:

(A10)

One can easily obtain the higher orders of correlations, however observing that all connected orders of events are reduced to the combinations of *pair-connected events*. For instance, we get for the second, third and fourth order fluctuations the respective average contributions:

(A11)

(A12)

(A13)

With *δ_ij_* type being the delta-Kronecker tensor. Next, for the quantum objects in question, *i*.*e*., for the classical path and connected Green function, the computing procedure consists of three major stages [52,53]: (i) Considering and solving the associate real time harmonic problem; (ii) Rotating the solution into imaginary time picture; (iii) Taking back the “free harmonic limit”, this way providing the respective results:

(A14)

(A15)

With the Heaviside step-function:

(A16)

With expressions (A14) and (A15) back in Equations (A8), (A11)–(A13) some imaginary-time integrals vanish, namely:

(A17)

while for the non-vanishing imaginary-time integrals appearing in Equation (A7), one obtains:

(A18)

(A19)

(A20)

(A21)

(A22)

(A23)

(A24)

With these, the earlier form Equation (A7) takes the particular expression for the propagator as in Equation (3), for *τ* = *ћβ* [30].

### Appendix 2: Gauss Type Relationships for Higher Order Integrals

One may apply the Gauss theorem successively for integrals of gradient of a given long range defined quantity (), successively as:

(A25)

(A26)

(A27)

(A28)

then specializing them for the attractive (bonding) potential to the working relationships:

(A29)

(A30)

(A31)

with the help of which the working Equation (5) for 4th order partition function is provided.
